# Environmental scan of family chart linking for genetic cascade screening in a U.S. integrated health system

**DOI:** 10.3389/fgene.2022.886650

**Published:** 2022-08-11

**Authors:** Cameron B. Haas, James Ralston, Stephanie M. Fullerton, Aaron Scrol, Nora B. Henrikson

**Affiliations:** ^1^ Kaiser Permanente Washington Health Research Institute, Seattle, WA, United States; ^2^ Department of Bioethics and Humanities, University of Washington School of Medicine, Seattle, WA, United States

**Keywords:** genomic medicine, cascade genetic testing, electronic health record, family health history communication, genetics, technology

## Abstract

**Background:** An alternative to population-based genetic testing, automated cascade genetic testing facilitated by sharing of family health history, has been conceptualized as a more efficient and cost-effective approach to identify hereditary genetic conditions. However, existing software and applications programming interfaces (API) for the practical implementation of this approach in health care settings have not been described.

**Methods:** We reviewed API available for facilitating cascade genetic testing in electronic health records (EHRs). We emphasize any information regarding informed consent as provided for each tool. Using semi-structured key informant interviews, we investigated uptake of and barriers to integrating automated family cascade genetic testing into the EHR.

**Results:** We summarized the functionalities of six tools related to utilizing family health history to facilitate cascade genetic testing. No tools were explicitly capable of facilitating family cascade genetic testing, but few enterprise EHRs supported family health history linkage. We conducted five key informant interviews with four main considerations that emerged including: 1) incentives for interoperability, 2) HIPAA and regulations, 3) mobile-app and alternatives to EHR deployment, 4) fundamental changes to conceptualizing EHRs.

**Discussion:** Despite the capabilities of existing technology, limited bioinformatic support has been developed to automate processes needed for family cascade genetic testing and the main barriers for implementation are nontechnical, including an understanding of regulations, consent, and workflow. As the trade-off between cost and efficiency for population-based and family cascade genetic testing shifts, the additional tools necessary for their implementation should be considered.

## Introduction

Cascade genetic screening is the practice of identifying at-risk relatives of individuals with known pathogenic genetic variants (Henrikson et al., 2020). Compared to population-based genetic testing, cascade genetic testing has been a historically more efficient and economical approach. In the United States, a person with actionable genetic test results is responsible for contacting their at-risk family members and communicating risk (Newson and Humphries, 2005). However, cascade testing communication is low and up to a third of at-risk relatives who may have actionable genetic findings go un-notified (Newson and Humphries, 2005; Griffin et al., 2020; Unger et al., 2020). This is thought to be in large part due to dependence on patients to share the information with family members (Henrikson et al*.*, 2019). Preliminary data suggests that patients who receive genetic testing are open to having their health system directly contact relatives who receive care in the same system to notify them of their potential risk (Mai et al., 2011; Henrikson et al., 2019).

Chart linkage is a functionality that enables connecting part or all of the electronic health records (EHRs) of different individuals. Family chart linkage is a potential strategy for facilitating information sharing needed for cascade testing (Hampel, 2016; Ohno-Machado et al., 2018; Caswell-Jin et al., 2019). If the presence of one person’s confirmed pathogenic variant could be noted in the EHR of their biologic relatives, care teams could use this information to recommend and order cascade genetic testing, potentially improving rates of both risk notification and cascade genetic testing. However, chart linkage and its implementation represent a substantial change from current practice and requires consideration of the clinical, technical, ethical, regulatory, and organizational implications, as well as patient and family preferences (Novak et al., 2013).

The Health Information Technology for Economic and Clinical Health (HITECH) Act in 2009 provided the catalyst for developing an incentive program for updating EHR systems to improve quality of care while maintaining compliance use the Health Insurance Portability and Accountability Act (HIPAA) Privacy Rule. The HITECH Act led to marked increases in structured and standardized documentation of family health history in EHRs, providing the potential for sharing family history information between family members using APIs.

We conducted an environmental scan of the current state of family chart linking bioinformatic tools and application programming interface (API), their limitations, and suggest an ethical framework for considering their clinical implementation. Along with identifying these tools, we sought to understand how policy- and decision-makers consider deciding whether to implement such a tool in clinical settings in the U.S.

## Methods

This study was reviewed and approved by the Institutional Review Board at the Kaiser Permanente Washington Health Research Institute.

### Guiding framework

**FIGURE 1:**
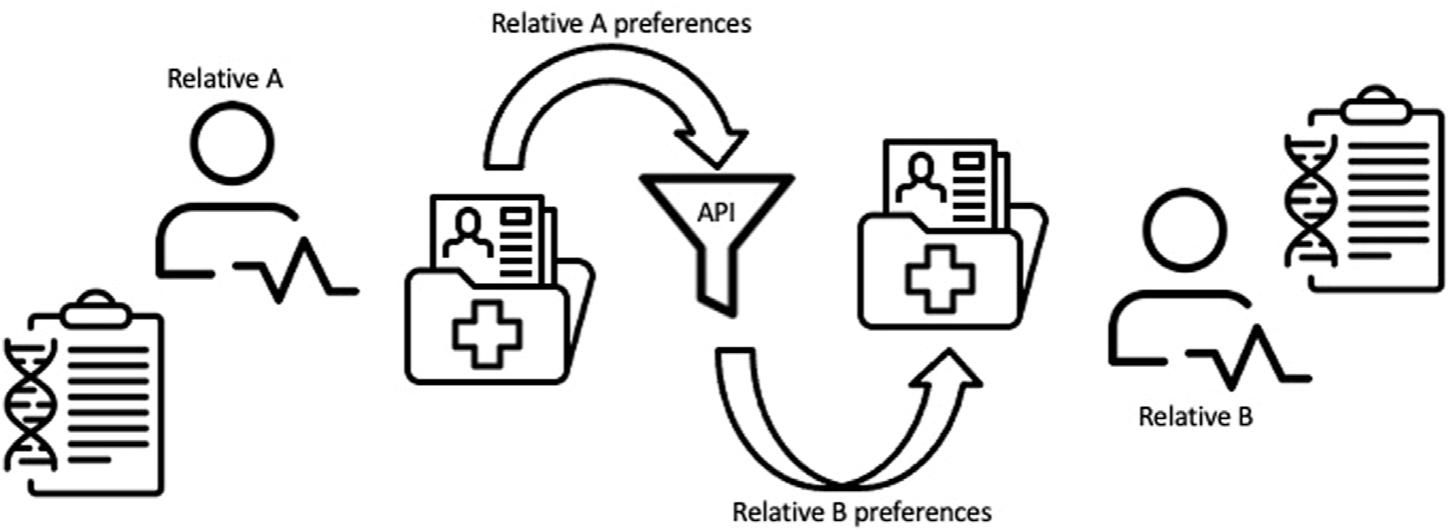
Conceptual framework for linking family health information between relatives within an EHR system.

We developed a conceptual framework to guide our environmental scan of tools for family chart linking between relatives in an EHR system (Figure 1) (Henrikson et al., 2021). In this representational model, the family health history or genetic information of Relative A as recorded in an EHR is processed through a tool before being modified and shared or transferred to the EHR of a consenting Relative B. The tool or API may be internal to the health care system EHRs or an external process that communicates through both relatives’ medical records (e.g., through a smartphone app that allows for bi-directional sharing of information between patients and their EHR). Output from the API is processed based on the preferences of Relative B and then used to inform clinical decision-making. Under this framework we assessed possible tools that might be used to achieve the process of sharing family health history between relatives in an EHR as envisioned in this model.

We were further guided in the development of the interview questions by the socio-technical model (Sittig and Singh, 2010), an 8-dimensional conceptual model of designed to identify sociotechnical challenges for health information technology, with the following domains: 1) *Hardware and software*, 2) *Clinical content*, 3) *Human computer interfa*ce, 4) *People*, 5) *Workflow and communication*, 6) *Internal organization features (e.g., policies, procedures, and culture)*, 7) *External rules and regulations*, 8) *Measurement and monitoring*.

### Electronic health record tools

We conducted an environmental scan to identify the current tools available for family chart linking and to understand the factors affecting clinical implementation (Choo, 2005). We used the Office of the National Coordinator for Health Information Technology (ONC Health IT) to identify certified Health IT products available from 2015 and later with active certification status and which met the certification criteria “170.315(A) (12): Family Health History”. We ended our tool search using ONC Health IT in February 2021. We focused our review on products developed for primary care practices, excluding those for specialized practices or intended exclusively for purposes related to prescriptions. For products with multiple versions listed we reviewed the latest version as of 5 February 2021. Additional tools were identified during key informant interviews and/or team reviews through the date of the last key informant interview, which was 14 March 2021.

We abstracted product names, functions and capabilities, interoperability, data structure and standards, recommended consents from system providers, and comparisons across systems into tables. We used concepts as described in Table 1 to characterize the minimal functional requirements for EHR suitability (Marsolo and Spooner, 2013), as well as the recommended consent process for each tool. Developers were contacted for additional commentary on the recommended consent process for the tools included in our analysis. We summarized notable features from these domains for each of the identified tools.

**TABLE 1:** Key concepts for reviewing electronic health record functional requirements for cascading genetic testing using family health history.

Concept	Description
*Structure*	Family history can be stored as structured or free-text data in the EHR system. While recent work in HL7 allows for standardized recording, ubiquitous adoption has been slow and unstructured documentation has been used in previous work applying natural language processing (Wang et al., 2017)
*Interoperability*	An increasingly valued component of EHR is the ability to share information between systems and healthcare providers. Interoperability allows for cooperative access and exchange between systems, with the goal to optimize communication. Linking between apps and EHR has been further facilitated by federal funding and the 21st Century Cures Act through lobbying efforts from SMART on FHIR, an open, free and standards-based API (Mandl and Kohane, 2009)
*Decision support process*	The primary utility of family health history in healthcare settings is to provide support for provider and patient in the shared decision-making process. Collection of family health history is the first step in reaching the potential health impact
*Updates to interpretation*	The dynamic nature of genetics requires reinterpretation and up-to-date information to identify clinically actionable findings. Most EHR systems are not designed to perform such tasks, as most family health history is considered static unless otherwise specified by patients
*Consent*	Describes the process for obtaining consent as recommended by the tool developer

### Key informant interviews

We identified potential key informants based on our objective of representing the perspectives of decision-makers managing healthcare system data and EHRs at public or private organizations and those who had and had not implemented family chart linking. We conducted key informant interviews to identify the considerations guiding decision-making and possible barriers to implementing family chart linking functionality in clinical systems. We used sequential non-independent review, receiving input from the study team after each source of data before proceeding to the next. Using an inductive approach, we developed frameworks for analyzing sources of information (Braun and Clarke, 2006).

The initial list of key informants included clinicians, industry or product developers, and researchers with expertise in family health history or genetic screening. We applied snowball sampling at the end of each interview to identify other relevant stakeholders. Key informants were invited by email to participate in an informational interview of 1–1.5 h durations and conducted using a video conferencing app (e.g., Zoom, Microsoft Teams). Financial incentives were not offered to key informants. The final key informant interview was conducted on 14 March 2021.

We developed a semi-structured interview guide for use during the interviews (Supplementary material). We referred to the domains of the socio-technical model as a guide for inclusion of questions for key informants. The interview guide was designed to meet the two following objectives: 1) to understand current use of family chart linking tools within the stakeholder’s system, and their choice of API (if currently using a tool) or their choice to forego using any API (if not currently using any tool); and 2) to understand the considerations or concerns for exemplary API for family chart linking from a systems perspective.

A single team member (CH) conducted interviews between February and March 2021. Interviews were recorded but not transcribed. Summarized notes were reviewed with participants at the end of each interview to verify points. The interviewer took extensive field notes during the interviews and wrote episode profiles of each interview. We used framework analysis, a rapid analysis technique where a priori codes are assigned based on the conceptual framework (Braun and Clarke, 2006). We iteratively summarized recurring considerations as they emerged, as related to dimensions of the socio-technical model. Findings were segmented into “users” and “non-users” of family chart linking API for a deductive approach of facilitators and barriers to implementing a tool in a clinical setting.

## Results

### Electronic health record tools

We identified six tools from five developers with functionalities related to family chart linking (Table 2). Search of the ONC Health IT database resulted in 181 unique products from 161 developers, (Supplementary Table S1). Reasons for exclusion included a focus on ambulatory services, pharmacy and prescriptions, optometry, and oncology or other specializations related to tertiary care. We explored online and publicly accessible resources for 45 products for mention of collecting either family health history or genetic information. We contacted 15 of those product developers with some online material regarding family health history for additional information and details related to tools available for family chart linking. We heard from one developer and accessed the remaining products based on available resources.

**TABLE 2:** Summary of tools and API with functionalities relevant to family chart linking and family health history sharing as of February 2021.

Product/Tool	Developer	Consent
Allow Clinicians to Copy Family History from a Patient’s Sibling	EpicCare^®^	Should consider same consent as Let Clinicians View or Edit Links to Family Members’ Chart (below). A system-wide setting can allow for an upper age limit for copying from a patient’s sibling
Let Clinicians View or Edit Links to Family Members’ Charts	EpicCare^®^	Expected that consenting policies will vary by organization. Each consenting policy could involve the following A new document type a relative digitally signs before their chart can be linked to other family members’ pedigrees. Clinicians would need to check for the form in the system before establishing the link
		A record of verbal consent from a relative
MyLegacy	Cerner	Patient enters data independently in a web-based questionnaire is SMART on FHIR compatible
myFHR	CareEvolution	myFHR allows you to share access to your health data with family and friends. Those that you have given access to will be able to use their myFHR app to view your health data such as lab results, current medications, and procedures and services. You can also request access to their health data
MeTree	Genomedical Connection	Patient initiated data collection and integration with medical records that support the SMART-FHIR standard
AncestryHealth kit	Ancestry (discontinued)	Consent is obtained at the time of purchase

Four of the included API were developed by EHR providers: EpicCare^®^, Cerner, and CareEvolution. One was a web-based program with Fast Healthcare Interoperability Resources (FHIR) standardizations to support EHR integration. The AncestryHealth kit was a shareable health report for clinicians and intended to be shared across family members based on direct-to-consumer (DTC) genetic test results, but the product has since been discontinued.

EpicCare^®^ provides two tools for relatives in the same EHR system. The first allows clinicians to copy structured family health history from one patient’s EHR to another’s. Transferring of this information is a one-time event and charts are not updated automatically between individuals. This functionality was created for scenarios in which newborn siblings are added to a health care system to eliminate the need to re-enter identical information. However, there is no upper age limit for which this tool can be applied. The second is the function to link individuals within an EHR system so that clinicians can view the charts of both family members. The links for this option are not bi-directional, meaning that the clinicians of Relative A could view the charts of Relative B but not necessarily vice versa (Table 2).

A separate approach, as exhibited by MyLegacy and myFHR, is a web-based program administered by a patient’s EHR system. The patients record family health history themselves and the data is collected in a standardized format (i.e., SMART on FHIR) to be viewed by the clinicians. This process allows a patient to share access to their information with others without giving direct access to their EHR. This option emphasizes external content for personalized decision-support. Similar to MyLegacy and myFHR, MeTree is a web-based app with an API to EHR systems.

Across all included tools, we found a lack of explicit guidance on the recommended consent process for sharing of family health history between relatives. EpicCare^®^ recommends healthcare organizations determine and implement consent policy and process necessary for sharing family history between relatives and recognizes that consenting policies may vary by organization.

### Key stakeholder interviews

We conducted five stakeholder interviews. Key informants (with abbreviated identifiers in parathesis) included a clinical geneticist from a not-for-profit medical group (CG), a population genetics researcher (R), a privacy management advisor for an integrated health care delivery system (P), a health services researcher (HS) and clinician with the U.S. Department of Veterans Affairs (VA), and a project manager for a private health IT developer (PM). The main considerations that emerged from summary of key informant interviews are described in Table 3.

**TABLE 3:** Key informant interview considerations for family chart linking and facilitating cascade genetic testing in electronic health record systems.

Sociotechnical Model Component	Considerations for Family Chart Linking
Hardware and software	• Interoperability between systems is possible and essential for widespread implementation
	• Paradigm shift toward shared information across the charts of family members
Clinical content	• Standardized data formats for clinical information required (e.g., genetic test results)
Human computer interface	• Third party apps where patients control flow of their information are possible alternatives to sharing within an EHR system
People	• Patient preferences for sharing genetic information with family members not well understood
Workflow and communication	• Paradigm shift away from physicians as gatekeepers of patient data
	• Large changes to workflow may be barriers to physicians already busy with competing demands
Internal organization features (e.g., policies, procedures, and culture)	• Competing demands for systems with high IT resource needs
	• Perceived evolution away from family-based genetic testing to universal screening
External rules and regulations	• HIPAA compliance
	• Procedures for patient and relative to consent to chart linking unclear
Measurement and monitoring	• Alternatives may be more favorable than chart linkage tools (e.g., maintain status quo; patient-controlled third party apps; universal genetic testing rather than family linkage with cascade testing)

#### Interoperability and standardization between systems was viewed as a critical technical requirement for family chart linkage

For an API that is external to a health care system EHR, emphasis on interoperability through standardized codes, “specifically HL7 (SMART on FHIR), would allow for better utility of family health history” (PM) Free-text or open comment fields have been historical means to collect history of family diseases, without a set standard of how the information should be collected or structured (PM). Another key informant was more concerned about the standardization of specific genetic test results, rather than the collection of family health history (CG). The key informant had concerns with how evolving interpretation of genetic test results could be standardized, as photocopies of paper results are still common in many EHR systems.

#### Clarity about HIPAA-related constraints was the primary barrier to implementation of family chart linkage programs

Nearly all participants mentioned HIPAA as the regulating factor when sharing family health history between patients, noting that it “governs when patient authorization is necessary” (P). However, one key informant felt that HIPAA was often over-interpreted and used as justification for lagging technology despite a lack of specific guidance in most circumstances (R).

#### Third-party and app-based solutions where patients manage data and sharing might have broader reach than linking individuals within the same EHR system

Some informants felt that there was little incentive for EHR systems to consider sharing of family health history between relatives since adult relatives do not commonly use the same EHR system (HS). One participant felt that we will need automated and patient-initiated solutions that are external to clinical EHR systems and that there would be more advancement in mobile apps with standardizing to SMART on FHIR (R). That key informant also suggested that HIPAA and consent might be easier to navigate in patient-initiated solutions rather than in a health care provider-initiated solution (R).

#### Paradigm shift in structure and directionality of EHR systems could be a solution for sharing family health information across relatives

One participant felt that a paradigm shift of the directionality of medical charts would need to shift from patient-provider to patient-patient in order to facilitate record linkages. They felt that clinicians are generally viewed as the gatekeepers of patient charts, even family health history, with the discretion to consult and share with other physicians as needed. Changing to a structure in which EHR data can be shared between patients would require a re-thinking of how health IT is structured (PM). Another paradigm shift would be thinking of family health history as a collective family chart rather than owned by a single individual. Patient records are thought of as individually owned, so a collective family record may change that mentality (HS). For this solution, the key informant envisioned a separate medical chart with family history information to which relative can link and share, rather than linking between family member charts directly and therefore restricting access to individual-level information. The key informant felt that a shared family record could also eliminate the concern for privacy regarding information not relevant to family members, as family-level data would be shared but individual-level data could still be restricted.

We noted additional comments that were mentioned by only a single key informant. For systems in which the health information technology is already lagging, automation of family health history and cascade genetic testing is low priority (HS). For primary care providers, for whom there is already a strain on resources and time, universal genetic testing presents a more simplified approach to identifying carriers of actionable genetic mutations (CG). We also note that these limitations to implementing family chart linkage and automated cascade genetic testing came from users of EHR systems, as opposed to non-patient-facing key informants. The consent process should be customizable to reflect the variation in comfort of patients to share their information with family members (P). A broad consent would be inadequate, particularly because future discoveries may change how to think about what we want to keep private (P). Some stakeholders felt that most EHR systems have to prioritize clinical support tools and interpretable genetic test results, and the ability to address the improvements required for cascade genetic testing will be obsolete once genetic testing becomes affordable for a universal testing approach (CG).

## Discussion

We conducted an environmental scan and key informant interviews to describe the current state of EHR-based or EHR-connected platforms for family chart linkage. Across evaluation of six chart-linkage tools and key informant interviews, we found that the technical capacity to build and implement family chart linkage exists. These technical aspects related to several domains in the socio-technical model, and most specifically show that the first domain, hardware and software, is not a main challenge facing the implementation of this functionality. However, several non-technical barriers may limit their adoption, including lack of clarity around HIPAA compliance issues; lack of guidance about optimal consent procedures for patient and relative consent to participate in chart linking; competing organizational demands; large changes to workflow required for implementation; and conceptual shifts in the current prevailing thought about the role of the physician and the structure and purpose of EHRs themselves. In the socio-technical model, these issues around the external rules and regulations appear to be a prominent challenge in the context of link family records. Additionally, in organizational settings facing competing demands for time and resources, alternative functionalities may exist that might appear more attractive, such as app-based health information sharing platforms where information flow is controlled by patients, not health care systems. The idea that family-based cascade genetic testing may soon be replaced by universal genetic screening was also noted.

Some record-linking functionality is currently available, but there are few tools for this specific purpose within the current EHR structure. Of the tools we identified, two were available through EpicCare^®^. EpicCare^®^ maintains over 30% of the EHR market share, particularly for large-scale health care systems in which generational family members are more like to be included and functions could be applied. Similarly, Cerner provides some functionality and represents a substantial market share of EHR systems, including the VA, where it is rare to have family members within the same health care system for which sharing of family health history would apply.

Interviews with key informants suggested that overcoming the limitation of sharing family health history between relatives of different EHR systems may best be solved through interoperability and third-party tools. Meaningful Use (MU), a result of the HITECH Act, outlines such incentives through the Center for Medicare and Medicaid System (CMS) and has been pivotal in creating incentives for electronic health record systems to improve their technology and functionality (Blumenthal and Tavenner, 2010). As of 2017, collection of family health history has been included as an optional component of MU, incentivizing health care systems to implement tools that allow for aggregating and reporting on family health history (Aziz et al., 2017). Stage 2 objectives of MU included the ability to record patient family health history in a structured data format and by 2013 over 85% of EHR systems had adopted this function (ONC, O. of the N. C. for H. I., 2014). A product of this emphasis on standardization has been wider adoption of Health Level Seven (HL7) International which has developed Fast Healthcare Interoperability Resources (FHIR), standards for API to allow the exchanging of EHRs. Building off of FHIR, SMART on FHIR was developed to transform EHRs into mobile-app based platforms. Standardization of family health history has been developed using HL7 FHIR but is still up for public comment of the current draft.

The future landscape of technology suggested to several of our key informants that third-party apps could be the most likely solution to sharing family health history between relatives for cascade genetic testing. A similar approach to using third-party tools for the interpretation of genetic DTC may be necessary to fill the current gap in needs (Nelson, Bowen and Fullerton, 2019), as well as for communicating genetic test results to at-risk family members (Haas et al., 2021). These mobile-based app approaches could provide additional tools for external content and shared clinical decision making. However, it is important to note that these types of patient-controlled solutions effectively put the onus of relative notification on patients. This is the current state of risk notification between relatives, where health systems have no direct role in risk notification, and has noted problems, including incomplete risk disclosure and patient burden (Henrikson et al., 2021). It is still unknown whether app-based notification would solve the known issues related to incomplete disclosure, which include; problematic family relationships, concerns about accuracy of patient-led disclosure, patient burden, and concern about distressing relatives. In that context, the use of third-party apps for risk sharing has to date shown limited promise (Haas et al., 2021).

Tools for collecting family health history have lagged compared to the advance in technology, with most still relying on paper-based forms and limited integration with EHR systems (Cleophat et al., 2018). The most widely used tools by genetic counselors, My Family Health Portrait (MFHP; freely available at www.familyhistory.hhs.gov) and Family Healthware (http://www.cdc.gov/genomics/about/family.htm), are not integrated into EHR systems (Feero, Bigley and Brinner, 2008). Qualitative work by Widmer et al. among genetic counselors found limited adoption of these tools because of their lack of integration into EHR systems (Widmer et al., 2013), seeking tools that were consistent (i.e., standardized), reduce repetitive questions, and improve clarity of clinical implications.

Non-intuitive or additional work arounds would likely put added burdens on clinicians. Primary care providers (PCP) continue to view their role to include the collection of family health history (Carroll et al., 2019). However, PCPs have often reported a lack of resources and tools for collecting and interpreting these details despite an expansion of genetic technology (Mikat-Stevens, Larson and Tarini, 2015; Carroll et al., 2019). As suggested by our interview with a clinical geneticist, the added burden of navigating cascade screening may point to universal genetic testing as a more feasible solution. While stakeholders interviewed in our study implied acceptability of universal genetic testing from their own perspective, future work should investigate patient perspectives with regards to implementing such an approach.

Several solutions to sharing family health history between relatives in an EHR system were suggested by key informants. However, these required major shifts in conceptualizing the purpose and directionality of patient records. Additionally, EHR systems are also structured within policies and regulations that would likely put constraints on changing this structure. Key informants felt that the limiting factor is the interpretation of regulations rather than the technological aspects, since the capacity to perform these tasks exists. However, one key informant felt that there was a tendency to be overly cautious with how regulations (e.g., HIPAA) are interpreted.

We acknowledge some limitations to this exploratory study. Our sample of key informants was small, and it is possible we missed some perspectives and settings, such as in oncology tertiary care. Due to the proprietary nature of the included tools, it is possible that there is guidance or information not publicly available to our team. Importantly, we were unable to interview patients and families given the limited scope of this project. Future research could explore patient and family thoughts on chart linking in more detail.

Limited progress has been made in terms of EHR functionality and interoperability. However, SMART on FHIR for smart devices may be filling the gap in needs for sharing of relevant information between family member but is limited the gatekeepers of patient EHR. More recent emphasis has been placed on improving clinical decision support and interpretation, benefits of which would not be confined to either a cascade screening or universal genetic testing approach (Carroll et al., 2019). Limited bioinformatic support has been developed to automate family cascade genetic testing. Though the technology to support family chart linkage is available, to date multiple substantial non-technical barriers exist to its implementation. The potential clinical benefits of these types of tools in facilitating cascade genetic testing and alleviating patient burden associated with patient-led risk disclosure could be explored in future research and contribute to the weighing of potential barriers.

## Data Availability

The original contributions presented in the study are included in the article/Supplementary Material, further inquiries can be directed to the corresponding author.
