# Supplementary material for: Environmental scan of family chart linking for genetic cascade screening in a U.S. integrated health system
Source: Front Genet. 2022 Aug 11;13:886650. doi: 10.3389/fgene.2022.886650 (PMC9403414; doi:10.3389/fgene.2022.886650)
Supplement: Supplementary file 2 [file DataSheet2.docx]

Interview Guide for family chart linking for genetic cascade screening key informant interviews

# Introduction:

We’re interested in understanding the current landscape of family chart linking in health care settings. As part of our study we hope to describe the current uptake of electronic health record tools that may facilitate the sharing of genetic information among family members within a health care system to aid in genetic cascade testing.

# Discussion questions:

I would first like to understand your role within your organization and your familiarity with collecting family health history in electronic health record systems.

1. Can you describe the organization your work for/with and your role in the organization?
2. What role does family chart linking play in your organization?
3. What direct experience have you had developing or using a family chart linking product?
   1. Are you aware of any technical issues with using a family chart linking tool within your organization? If so, please describe them.
   2. What information is shared between family members?
   3. What is the clinical content that physicians see?
4. What are the potential benefits of family chart linking?
5. What metrics would you be interested in seeing to measure or monitor the impact of family chart linking?
6. What are the potential barriers to implementing family chart linking in your or other organizations? (probe for leadership, legal, patient, technical, cultural, resources)

# Tools for family chart linking

Some tools currently exist that would facilitate family chart linking. For example, tools that allow clinicians to enter medical charts of their patient's relatives currently exist. This tool recommends obtaining consent prior to implementing this linking but leaves the form of consent up to the user.

1. What process do you believe would be necessary to enable family chart linking in your organization? How would you decide on the appropriate format for obtaining consent?
2. Are there similar processes currently implemented in your organization that require obtaining patient consent?

Another tool currently available allows clinicians to copy the family history of siblings directly between patients. While there is no default age requirement that would allow the sharing of family health history between adult patients, the tool was intended for minors and children without any existing family health history recorded in their charts.

1. Would you consider putting an age restriction on this functionality? If so, what would it be?
2. Would there be utility in expanding this functionality beyond sibling relationships?
3. What consenting process would your organization consider? How might this circumstance differ from the previously mentioned functionality (entering relatives’ medical charts)?

# Conclude

1. What do you think the future of such family chart linking tools will look like in 5-10 years?

I appreciate you taking some time today to meet with me and discuss this topic. It’s a quickly evolving area of research and we’re trying to gain as much insight on it as possible.

1. Who else is doing work in this area? Who else should I talk with?
